# Society Meetings

**Published:** 1918-04

**Authors:** 


### SOCIETY MEETINGS

**Brief reports and announcements of meetings of societies of Western New York. and those of general scope, are requested from Secretaries. Copy should be on hand the fifteenth of the month. Full transactions will be published at cost of composition.**

**Rochester Academy of Medicine**; meeting held March 13. Dr. William M. Brown read a paper on "The Role of the Liver, in the Toxemia of Pregnancy."

